# Proceedings of the Simulation Congress 2019: Liege, Belgium. 28 March 2019

**DOI:** 10.1186/s13690-019-0359-8

**Published:** 2019-07-10

## Session 1: Simulation in obstetrics

### A1 Implementation of an OSCE in the initial training of nurses and midwives in Morocco: assessment of skills and perception

#### Omaima Changuiti, Samia Chergaoui, Abdellah Gantare, Abdelghafour Marfak, Abderraouf Hilali, Ibtissam Youlyouz-Marfak

##### Laboratoire des sciences et technologies de santé (STS), Université Hassan 1^er^, Settat, Morocco

###### **Correspondence:** Omaima Changuiti (o.changuiti@uhp.ac.ma)

**Background**

The main objective of healthcare training is to foster the development of clinical skills in students according to each level of study. The differences of experiences, the teaching methods and the use of irrelevant forms of assessment are obstacles in achieving this goal. There is a need to use OSCE (Objective Structured Clinical Examination) as an assessment tool to objectively and equitably evaluate the clinical skills of health sciences students [1]. This tool has become widely used in the world of medical education, specifically in training involving simulation as a valid learning method [2]. But there is a flagrant lack in the paramedical field.

**Materials and methods**

The present study reports the process of setting up the OSCE in nursing and midwifery training at the Hassan 1 University. Students are assessed on two stations that follow a logical progression suitable for each level of study. We elaborated adapted scenarios according to the objectives of each station. We developed the assessment grids which were then validated by the specialty teachers according to the objectives of each station. Student perceptions were collected through a satisfaction questionnaire delivered at the end of each session. The results of the grids are scored and analysed using the Excel software, and the analysis from the questionnaire was carried out using SPSS software.

**Results**

The results of the assessment grids clearly clarified the level of success and failure for each student, which were different according to each level of study. Based on the analysis of the questionnaires, it was found that most students (94%) were satisfied with the assessment and appreciated the learning experience.

**Conclusions**

The integration of the OSCE in the paramedical educational program at the Hassan 1 University in Morocco is needed to properly assess the clinical skills of students.

**References**

1. Harden RM, Gleeson FA. Assessment of clinical competence using an objective structured clinical examination (OSCE). Med Educ, 1979; 13(1), 41-54.

2. AlOsail A, AlShiekh M, AlSaid A, AlOsail E, AlGhamdi M, Alhawas A, Albahussain A, Aldajani A. Objective Structured Clinical Examination as an Assessment Method for Undergraduate Medical Students. International Journal of Medical Science and Public Health. 2015; 4(2): 192-198.

### A2 The effect of simulation on clinical and non-technical competences acquisition of midwifery students in Morocco

#### Samia Chergaoui, Omaima Changuiti, Abdelghafour Marfak, Abdellah Gantare, Abderraouf Hilali, Ibtissam Youlyouz-Marfak

##### Laboratoire des sciences et technologies de santé (STS), Université Hassan 1^er^, Settat, Morocco

###### **Correspondence:** Samia Chergaoui (samia.chergaoui@uhp.ac.ma)

**Background**

Simulation is, more than ever, an indispensable teaching method for all health professionals in training and continuing training [1]. It helps acquire the technical skills (procedural practices, acts) and non-technical skills (verbal and nonverbal communication, teamwork, leadership, etc.). Then, simulation offers students the opportunity to learn from their mistakes without adverse physical and psychological consequences for them or the patients [2]. This study aims at evaluating the interest of the simulation, and its impact on the student midwives in initial training.

**Materials and methods**

The study involved 25 volunteers, all first-year students in midwifery sciences, who were divided into 2 groups after a lecture given by experts. Group 1 received a pre-test and then a simulation session followed by a post-test the same day. Group 2 (control group) received the same pre-test as Group 1 then was asked to review the lecture and after a week they received a post-test I, which was followed by a simulation session the same day and then after one week they received the post-test II. Pre- and post-tests contained 11 questions scored according to the importance of each of them in relation to the technical act concerned by the study.

**Results**

In Group 1, we found that the average score increased from 14.33 to 21.11 between pre- and post-test (p-value =0.03). In Group 2, there was a slight increase between pre- and post-test scores, is 13.08 to 16.75. However, after the simulation session, the average of the post-test II increased to 20.58.

**Conclusions**

Finally, a clear improvement has been noticed on a technical and relational level. Simulation included effectiveness and efficiency with taking the patient's safety into consideration.

**References**

1. Granry JC, Moll MC. Etat de l’art (national et international) en matière de pratiques de simulation dans le domaine de la santé. Dans le cadre du développement professionnel continu (DPC) et de la prévention des risques associés aux soins. Paris : Haute Autorité de Santé; 2012.

2. Lassalle V., Berton J., Bouhours G. et al. Medical paediatric simulation: a European Survey. Ann Fr Anesth de Réanim. 2009;28:628-633

### A3 Simulation training improves health care providers’ skills in the management of obstetrical complications in Democratic Republic of Congo

#### Vicky Lokomba^1^, Andy Mbangama^1^, Guy Mukumpuri^2^, Aline Mulunda^3^

##### ^1^Department of Obstetrics and Gynaecology, University Clinics of Kinshasa, Kinshasa, Democratic Republic of Congo; ^2^Maternal Health Division of the National Reproductive Health Program, Kinshasa, Democratic Republic of Congo; ^3^Superior Institute of Medical Technics, Kinshasa, Democratic Republic of Congo

###### **Correspondence:** Vicky Lokomba (vlokomba@yahoo.fr)

**Background**

The maternal mortality rate (MMR) in DR Congo, about 846 deaths per 100.000 live births, is one of the highest in the world. This is more dramatic in rural areas. From analysis of maternal death surveillance, it appears that, among many factors, lack of effective care is the major cause of maternal death. We aimed to assess the knowledge and skills of healthcare providers in the emergency obstetrical care (EmOC) management before and after simulation training.

**Materials and methods**

Using low-technology manikins, 50 doctors and 72 nurses from referral hospitals and health centres of high MMR areas received a 15-days training on obstetrical complications management. The first part of the program consisted of interactive lectures and watching video of basics in the delivery room (46 hours): systematic monitoring, infection prevention, management of obstetrical complications and maternal and neonatal resuscitation. The simulated practice training part used low fidelity obstetric and new-born anatomic models and interactive workshops (50 hours). Pre- and post-training evaluation was conducted with a multiple-choice questionnaire for theoretical knowledge and a checklist for EmOC skills. T-test was performed to ascertain whether the EmOC training intervention was effective (p < 0.05)

**Results**

Training resulted in a significant improvement in both theoretical and practical skills of health care providers. The mean theoretical knowledge score increased between before and after training from 38.14% ± 15.2 to 64.38% ± 15.1 respectively (p < 0.001) and from 18.02 ± 9.4 to 60.3 ± 9.1 (p < 0.001) for practical skills which improved in all competences (p < 0.001). Changes for both aspects were similar between doctors and nurses.

**Conclusions**

Simulation training of perinatal care providers in low resources conditions improved theoretical and practical skills, even with low-technology manikins. This strategy could decrease the very high rate of obstetrical complications in DR Congo.

## Session 2 : Evidence-based simulation

### A4 From simulation to dissemination: effects of the “school saves lives” intervention

#### Alexandre Mouton^1^, Adrien Closter^1^, Lucien Colard^1^, Manon Collin^1^, Simon Verdonck^2^, Denis Ulweling^2^, Marc Cloes^1^

##### ^1^Sport Sciences Department, University of Liege, Liege, Belgium; ^2^Francophone Belgian Lifesaving Association, Louvain-la-Neuve, Belgium

###### **Correspondence:** Alexandre Mouton (alexandre.mouton@uliege.be)

**Background**

At the time being, 90 percent of people who experience an out-of-hospital cardiac arrest still die [1]. The European Resuscitation Council (ERC) states that teaching cardiopulmonary resuscitation (CPR) and the use of automated external defibrillator (AED) at school, defined as basic life support (BLS), would have a significant impact on survival rate [2-3]. Thanks to their training, physical education (PE) teachers are ideally placed to learn BLS to their students [4-5]. The aim of the study was to evaluate the relevance and the impact of a BLS sequence taught by PE teachers on theoretical knowledge and practical life-saving skills of the students.

**Materials and methods**

Twenty-one secondary school PE teachers were recruited and trained to a specific BLS cycle adapted to each teaching level (n=3). Students learned the BLS protocol during 6 sessions of PE with hands-on application on training manikins, AEDs, and innovative interactive videos. Students’ knowledge of the BLS protocol was assessed by an open-ended questionnaire at baseline (T0), after the intervention (T1), and after a follow-up period of 3 months (T2). Practical application of the BLS protocol was assessed on a manikin measuring CPR performance at T1 and T2.

**Results**

In each teaching level, students (1^st^: 10.7±0.8 years, n=186; 2^nd^: 14.5±0.9 years, n=112; 3^rd^: 17.1±0.8 years, n=307) demonstrated significant improvements of knowledge of the BLS protocol at T1 (p<.0001) that remained stable at T2. Second- and third-cycles students were able to perform chest compressions close to the international recommendations [6] at T1 (respectively 37.3±6.5mm; 43.3±7.7mm) and T2 (respectively 36.8±7.5mm; 41.4±7.6mm). More than 80% of the students felt able to help a victim of cardiac arrest at T1 and T2.

**Conclusions**

The BLS sequence led to encouraging improvements of knowledge, abilities, and confidence of the students. PE teachers felt valuated and able to contribute autonomously to this major public health challenge.

**References**

1. Berdowski J, Berg RA, Tijssen JG, et al. Global incidences of out-of-hospital car-diac arrest and survival rates: Systematic review of 67 prospective studies. Resuscitation. 2010;81:1479-1487.

2. Plant N, Taylor K. How best to teach CPR to schoolchildren: a systematic review. Resuscitation. 2013;84:415-421.

3. Conolly M, Toner P, Connolly D, McCluskey DR. The “ABC for life” programme - teaching basic life support in schools. Resuscitation. 2007;72:270-279.

4. Colquhoun M. Learning CPR at school – everyone should do it. Resuscitation. 2012;83:543-544.

5. Mouton A., Laurent C., Collin M., Verdonck D., Ovart D., Ulweling D., Cloes M. “Dare to save a life at school”: Implementation of a CPR+AED sequence in the PE curriculum. Resuscitation. 2017;118S:e43-e90.

6. European Resuscitation Council. European Resuscitation Council Guidelines for Resuscitation. 2015. Resuscitation. 2015;95:1-312.

### A5 Impact of simulation training for intravenous medication administration safety: a randomised controlled study

#### Jean-Christophe Servotte^1,2^, Alexandre Ghuysen^1,2^, Konstantinos Gasteratos³, Nadia Dardenne^1^, Isabelle Bragard^1,2^

##### ^1^Department of Public Health Sciences, University of Liege, Liege, Belgium; ²Center of Medical Simulation, University of Liege, Liege, Belgium; ³Queen Mary University of London, London, England

###### **Correspondence:** Jean-Christophe Servotte (jcservotte@uliege.be)

**Background**

Medication administration safety and prevention of errors are key components of nurses’ day-to-day care [1]. The prevalence of intravenous medication administration errors is persistently high [2]. Nursing students and newly graduated nurses lack knowledge in drug calculation and skills for safely administrating medications [3-4]. Simulation-based training (SBT) has been advocated to cope with this issue and enhance learning in this domain [3,5].

This randomised controlled study compared the impact of a 3-hours standardised patient SBT with a 4-hours traditional course including a 2-hours theoretical course and a 2-hours workshops focusing on medication preparation. Impact assessment included long-term nursing students’ knowledge and skills for intravenous medication administration in skill lab as well as on the real clinical setting in ICU.

**Materials and methods**

Nursing students (n=99) were assigned to a control group (CG, n=50) which followed the 4-hours traditional course or to an experimental group (EG, n=49) which beneficiated from an additional 3-hours SBT. Participants were assessed at three different times: at pre-test (T0), one month later (T1) and nine months later (T2). Knowledge focusing on medication administration safety principles was measured with a 10-items questionnaire. The Medication Administration Safety Assessment Tool (MASAT) [6] assessed medication administration skills. Two blinded experts rated the students with MASAT. Students were also assessed with MASAT during their clinical apprenticeship in intensive care unit (T3).

**Results**

We found no statistical differences between groups at T0. At T1, T2 and T3, EG significantly increased knowledge (p < 0.001, at each times) and skills (p < 0.001, at each time) as compared with the CG.

**Conclusions**

A 3-hours SBT can potentially offer a persistent transfer of learning on clinical practice. This study suggests that intravenous medication administration safety simulation is more effective than traditional pedagogical method. The effect seems to be persistent.

**References**

1. Miller K, Haddad L, Phillips K. Educational strategies for reducing medication errors committed by student nurses: a literature review. Int J Health Sci Educ, 2016;3(1):1–17.

2. Barker KN, Flynn EA, Pepper GA, Bates DW, Mikeal RL. Medication errors observed in 36 health care facilities. Arch Intern Med, 2002;162(16):1897–1903.

3. Mariani B, Ross JG, Paparella S, Allen LR. Medication safety simulation to assess student knowledge and competence. Clin Simul Nurs, 2017;13(5):210-216.

4. Molloy J. Reinforcing medication administration through student-directed simulation. Teach Learn Nurs, 2017; 12:307-308.

5. Jarvill M, Jenkins S, Akman O, Astroth KS, Pohl C, Jacobs PJ. Effect of simulation on nursing students’ medication administration competence. Clin Simul Nurs, 2018;4:3-7

6. Goodstone L, Goodstone MS. Use of simulation to develop a medication administration safety assessment tool. Clin Simul Nurs, 2013;9(12), e609-e615.

### A6 Breaking bad news in the emergency department: impact of training through e-learning and role-play

#### Pauline Van Ngoc^1,2^, Jean-Christophe Servotte^1,2^, Anne-Sophie Nyssen^3^, Alexandre Ghuysen^1,2^, Isabelle Bragard^1,2^

##### ^1^Department of Public Health, University of Liège, Liège, Belgium; ^2^Centre of Medical Simulation, University of Liège, Liège, Belgium; ^3^Laboratoire d'Ergonomie Cognitive et d'Intervention au Travail, University of Liège, Liège, Belgium

###### **Correspondence:** Pauline Van Ngoc (pauline.vanngoc@uliege.be)

**Background**

In the emergency department (ED), breaking bad news (BBN) is part of physicians’ daily tasks and requires strong communication skills [1]. In this unpredictable context, BBN is frequently poorly delivered and causes anxiety for both physicians and patients [2]. This study examines the efficacy of a short BBN training in the ED on communication skills, sense of competence and stress. This BBN training is composed of an e-learning course and role-play simulations. Therefore, this study compares two conditions: clinical practice and BBN training compared to clinical practice alone.

**Materials and methods**

Medical students in their third year of master (n=35) were randomly assigned to either an experimental group (EG; n=14) that received the short BBN training (e-learning and role-play) between two assessment phases, or a control group (CG; n=21) that received the training after the two phases. Assessments included a BBN simulation with standardised patients (SP). A rater analysed communication skills using the SPIKES Competence Form [3], the BBN Assessment Schedule (BAS) [4] and the Health Communication Assessment Tool [5]. Visual analogue scales were used to assess the trainees’ stress and SP’s satisfaction regarding the trainees’ performance. Finally, the sense of competence was evaluated using a 5-point Likert scale questionnaire.

**Results**

The improvement of communication skills (BAS, p<.05) and BBN steps respect (SPIKES, p<.001) was statistically significant in the EG, compared to CG. Results showed a statistically significant increase of SP’s satisfaction in EG compared to CG (p<.05). Both groups reported a statistically significant sense of competence’s increase (p<.01) and stress reduction (p<.05).

**Conclusions**

Both groups perceived an improvement of their communication and stress level, but only the group that had received the simulation training showed a real improvement of performance and better SP’s satisfaction. It thus seems essential to include this kind of non-technical skills training in the medical curriculum.

**References**

1. Chumpitazi CE, Rees CA, Chumpitazi BP, Hsu DC, Cara B, Lorin MI. Creation and Assessment of a Bad News Delivery Simulation Curriculum for Pediatric Emergency Medicine Fellows. 2016;8:1–13.

2. Nancy C, Larson J, Scofield S, Sourkes B, HJ C. Hospital staff and family perspectives regarding quality of pediatric palliative care. Pediatrics. 2004;114:1248–52. doi:10.1097/01.CCM.0000127262.16690.65.

3. Park I, Gupta A, Mandani K, Haubner L, Peckler B. Breaking bad news education for emergency medicine residents: A novel training module using simulation with the SPIKES protocol. J Emerg Trauma Shock. 2010;3:385–8. doi:10.4103/0974-2700.70760.

4. Miller SJ, Hope T, Talbot DC. The development of a structured rating schedule (the BAS) to assess skills in breaking bad news. Br J Cancer. 1999;80:792–800.

5. Campbell SH, Pagano MP, O’Shea ER, Connery C, Caron C. Development of the health communication assessment tool: Enhancing relationships, empowerment, and power-sharing skills. Clin Simul Nurs. 2013;9:e543–50. doi:10.1016/j.ecns.2013.04.016.

## Session 3 : Virtual simulation

### A7 A virtual patient to enhance physicians training in breaking bad news in oncology

#### Manon Goosse^1^, Iness Ortiz^1^, Nicole Barthélemy^2^, Michèle Guillaume^1^, Alexandre Ghuysen^1,2,3^, Guy Jérusalem^4^, Isabelle Bragard^1,2,3^

##### ^1^Public Health Department, University of Liege, Liege, Belgium; ^2^Radiotherapy Department, University Hospital of Liege, Liege, Belgium; ^3^Centre of Medical Simulation, University of Liege, Liege, Belgium; ^4^Medical Oncology Department, University Hospital of Liege, Liege, Belgium

###### **Correspondence:** Manon Goosse (manon.goosse@uliege.be)

**Background**

As inadequate Breaking Bad News (BBN) negatively impacts both patients [1,2] and physicians [3], training physicians to BBN is essential [4]. So far, training on standardised patients have shown positive results but require important resources [5]. Studies provide encouraging results regarding virtual patients’ cost-effectiveness (VP) [6, 7, 8]. However, these underline difficulties: representation of VPs nonverbal cues and lack of immediate feedback [6, 7]. Therefore, we aim at providing a VP overcoming these difficulties.

**Materials and methods**

VP’s development includes two sections: (1) VP’s nonverbal emotional expression, (2) VP and learner’s verbal statements. Based on micro-facial expressions defined by Ekman (2003) [9], a database of faces’ pictures, illustrating anger, sadness and shock, has been identified and validated by a sample of 24 participants from different education background. For each picture’s validation, 50% of the sample had to agree on the emotion’s type. This process was repeated twice to obtain the final sample of pictures. Then, the dialogues were developed in collaboration with oncologists and experts in communication based on the BBN SPIKES model [10, 11]. Decision trees structuring these verbal statements have been created. During each interaction, learners will have to choose orally between 3 possibilities of answers and will receive immediate feedback, through the variation of VPs’ emotion or speech, according to their choices’ adequacy.

**Results**

Regarding the first section, 23 faces’ pictures were validated to create three emotional animation loops (for anger, sadness and shock). For the second section, five successive decision trees allow covering the 6 steps of SPIKES model. Each tree includes 28 to 36 programmed utterances for the physician and 27 to 28 for the patient.

**Conclusions**

The creation of VP is still in progress. A sketch will be available in March 2019. We plan on testing the VP with medical students to verify that it addresses the issues reported in literature. The learning impact will be tested afterwards.

**References**

1. Fang F, Fall K, Mittleman MA, Sparén P, Ye W, Adami HO, Valdimarsdóttir U. Suicide and Cardiovascular Death after a Cancer Diagnosis. N Engl J Med. 2012; 366, 1310-1318

2. Zachariae R, Pedersen CG, Jensen AB, Ehrnrooth E, Rossen PB, von der Maase H. Association of perceived physician communication style with patient satisfaction, distress, cancer-related self-efficacy and perceived control over the disease. **Br J Cancer.** 2003; 88, 658-665

3. Bragard I, Etienne AM, Libert Y, Merckaert I, Razavi. Efficacy of a communication and stress management training on medical residents’ stress to communicate, self-efficacy and burnout: A randomized controlled study. **J Health** Psychol. 2010; 15: 1075-1081

4. Dosanjh S, Barnes J, Bhandari M. Barriers to breaking bad news among medical and surgical residents. Med Educ. 2001;35(3):197–205.

5. Ochs M, Blache P. Virtual Reality for Training Doctors to Break Bad News. In: European Conference on Technology Enhanced Learning. 2016. p. 466–71.

6. Kava BR, Andrade AD, Marcovich R, Idress T, Ruiz JG. Communication Skills Assessment Using Human Avatars: Piloting a Virtual World Objective Structured Clinical Examination. Urol Pract. 2017;4(1):76–84.

7. Andrade AD, Bagri A, Zaw K, Roos BA, Ruiz JG. Avatar-Mediated Training in the Delivery of Bad News in a Virtual World. J Palliat Med. 2010; 13: 1415‑1419

8. Kron FW, Fetters MD, Scerbo MW, White CB, Lypson ML, Padilla MA, … Becker, DM. Using a computer simulation for teaching communication skills: A blinded multisite mixed methods randomized controlled trial. **Patient** Educ Couns. 2017; 100: 748‑759

9. Ekman P. Emotions revealed: Recognizing Faces and feeling to improve communication and emotional life. New York, NY: Times Books; 2003.

10. Baile WF. SPIKES-A Six-Step Protocol for Delivering Bad News: Application to the Patient with Cancer. The Oncologist. 2000; 5, 302‑311.

11. Buckman R. S’asseoir pour parler. Paris, France : Masson; 2001.

### A8 Development of virtual reality tools for urgent medical care: the ORVAMU project

#### Stéphane Grade, Michel Vergnion

##### Ecole provinciale d’aide médicale urgente (EPAMU), Seraing, Belgium

###### **Correspondence:** Stéphane Grade (stephane.grade@hepl.be)

**Background**

Simulating emergency situations in virtual reality (VR) offers great opportunities for supporting the training of medical professionals. These VR applications have been proved to improve learning outcomes for different procedures in various medical domains [1,2]. Several elements (environments, avatars), once put together, provide excellent training grounds. Research suggests that trainees beneficiating from VR simulation reinforce their decision-making skills in settings mimicking realistic situations [1,2]. Accordingly, we developed VR applications to train and evaluate skills like medical triage and bleeding control.

**Materials and methods**

The development of these VR applications is based on a literature review, other existing applications and documents of the Belgian Federal Ministry for Public Health. Moreover, the development team participated in several live simulation exercises to get a global overview and try to digitalise key aspects of those simulations. Finally, the learning outcomes of the VR applications were clearly specified such as triage and damage control.

**Results**

Resulting from this, 30 virtual victims were created, each with their own parameters (e.g., respiratory frequency, consciousness level) and some of them with specific wounds. Three different environments (parking, restaurant, school) have been set up, where the victims are scattered in different locations of the environment. Finally, the immersive simulation provides the user with the ability to perform evaluations (i.e., getting victim’s parameters) and interventions (e.g., tourniquet, triage coloured tags). The system records the choices/actions performed by the user to give them feedback on their performances during the simulation or the debriefing.

**Conclusions**

We plan on comparing traditional training (lectures) to a VR training session in order to assess the added value of our VR tools on learning. Further uses like evaluating user’s skills (novice vs. experts) or comparing the emotional impact of different scenarios (infant vs. adult victims) will also be considered.

**References**

1. McGrath JL, Taekman JM, Dev P, Danforth DR, Mohan D, Kman N, ... & Talbot TB. Using virtual reality simulation environments to assess competence for emergency medicine learners. Academic Emergency Medicine. 2018; 25(2): 186-195.

2. Ingrassia PL, Ragazzoni L, Carenzo L, Colombo D, Gallardo AR, & Della Corte F. Virtual reality and live simulation: a comparison between two simulation tools for assessing mass casualty triage skills. European Journal of Emergency Medicine. 2015; 22(2): 121-127.

### A9 Evaluation of surgical simulation in temporal bone surgical radio-anatomy learning

#### Florence Rogister^1^, Caroline Salmon^1^, Alexandre Ghuysen^2^, Pierre Bonnet^3^, Séverine Camby^4^, Philippe Lefebvre^5^, Anne-Lise Poirrier^5^

##### ^1^Department of Otorhinolaryngology, University Hospital of Liège, Liege, Belgium; ^2^Department of Emergency Medicine, University Hospital of Liège, Liege, Belgium; ^3^Department of Anatomy, University Hospital of Liège, Liege, Belgium; ^4^Department of Otorhinolaryngology, University Hospital of Liège, Liege, Belgium; ^5^Department of Otorhinolaryngology, University Hospital of Liège, Liege, Belgium

###### **Correspondence:** Florence Rogister (frogister@student.uliege.be)

**Background**

Lack of data regarding the efficiency of simulation-based teaching, in particular high-fidelity virtual reality, constitutes a major drawback for its implementation in medical training. However, it provides a structured, safe and supportive environment to become familiar with complex anatomy and practice surgical skills. We aimed to evaluate high-fidelity virtual reality simulation in learning of temporal bone radio-anatomy during ENT residency.

**Materials and Methods**

15 Belgian otorhinolaryngology residents completed 5 sessions of simulation in antro-mastoidectomy using the VOXEL-MAN Tempo® surgical simulator. Technical mistakes and surgical parameters were recorded. Before and after the training period, residents completed an online test on temporal bone radiological anatomy (*http://www.radioanatomie.com**).* Pre- and post-simulation scores were compared as primary endpoint by non-parametric Wilcoxon test using RCmdr (https://www.r-project.org). As secondary endpoint, the residents’ surgical skills following simulation training were blindly assessed on cadaveric human temporal bones using a reproducible scale by senior’s otologist surgeons of our department. Correlation between the radiological testing and the dissection scale were evaluated by Spearman regression. Finally, the trainees completed a survey on the device itself.

**Results**

Performances on radiological testing significantly increased with a mean improvement of 28 ± 12.12 (Wilcoxon p = 0.0011). The surgical results on cadaveric specimens were not correlated to surgical simulation parameters. Higher results on radiological testing were associated with higher scores on dissection scale, suggesting that the subjects who succeeded better at dissection were those who knew anatomy best. Among the residents, 80% perceived this tool as suitable for early surgical education, and 84.6% queried further information on simulation techniques. 100% of trainees would integrate this tool within their learning of temporal bone's radiological and surgical anatomy.

**Conclusions**

The high-fidelity virtual reality simulator has improved temporal bone anatomy teaching, and specifically the knowledge of the anatomy of the temporal bone radiological.

**Acknowledgements**

No conflict of interest.

## Session 4 : Non-technical skills and human factors

### A10 Simulation-based training development to strive for excellence in radiation oncologist’s education

#### Nadège Dubois^1,2^, Séverine Cucchiaro^2^, Selma Ben Mustapha^2^, Michael Noel^2^, Willy Ruyange^2^, Alexandre Ghuysen^1^, Isabelle Bragard^1^, Philippe Coucke^2^

##### ^1^Center of Medical Simulation, University of Liège, Liège, Belgium; ^2^Radiotherapy Department, University Hospital of Liège, Liège, Belgium

###### **Correspondence:** Nadège Dubois (nadege.dubois@chuliege.be)

**Background**

The heterogeneity of radiotherapy (RT) support worldwide has led the International Atomic Energy Agency to claim for recommendations regarding hospital infrastructures and staff training programs [1]. Following these observations, we aim at setting up a training curriculum for RT through an inter-regional project within the greater region (NHL-ChirEx 3476). Such a curriculum will focus on standardised care and prompt cross-border exchanges of practices and knowledge. It will particularly focus on safety, patient-centred care.

**Materials and methods**

We initially conducted a retrospective incident analysis within the RT department focusing on reported adverse events (AE) from September 2017 to November 2018. AE were inventoried then classified according to their origin. Secondly, we will set up survey evaluating expectations and needs regarding the training of residents in RT in order to best target their learning wishes. Based on these two steps, the curriculum will be developed and implemented integrating technology-enhanced learning tools such as: e-learning, simulation-based learning and virtual reality immersion, especially the Virtual Environment for Radiotherapy Training or VERT^® [^2]. This environment provides a safe clinical experience and allows students to improve their practical patient care, but also their linear accelerator treatment administration without any risk.

**Results**

Results from step 1 reveal that most AE had no (47,7%) or slight (40,4%) degree of severity [3]. Considering the nature of the events, we used a classification based on the patient's process during treatment and organisational factors [4]. Out of 386 reported AE, problems related to the organisation and information transfer represent more than one third of AE (respectively 21 and 15,5%).

**Conclusions**

Further results could help us pick out the best pedagogic strategy to adopt depending on the skills to be developed. Future evaluation of this programme could lead to integration of more simulation-based training in education programmes and continuing education within institutions.

**Acknowledgements**

This abstract is presented on behalf of an Interreg funded project (Grant N°043-1-01-125).

**References**

1. International Atomic Energy Agency. Setting Up a Radiotherapy Programme: Clinical, Medical Physics, Radiation Protection and Safety Aspects. Vienna; 2008.

2. Phillips R, Ward JW, Page L, Grau C, Bojen A, Hall J, et al. Virtual reality training for radiotherapy becomes a reality. Stud Health Technol Inform. 2008;132:366–71.

3. Autorité de sûreté nucléaire. Guide d'auto-évaluation des risques encourus par les patients en radiothérapie externe. Paris; 2009.

4. World Health Organization. Radiotherapy risk profile. Switzerland; 2008.

### A11 School-based disaster preparedness: are non-technical skills taught?

#### Méryl Hajaoui^1^, Alexandre Ghuysen^1,2^, Isabelle Bragard^1,2^

##### ^1^Public Health Department, University of Liege, Liege, Belgium; ^2^Centre of Medical Simulation, University of Liege, Liege, Belgium

###### **Correspondence:** Méryl Hajaoui (meryl.hajaoui@uliege.be)

**Background**

The constant increase in crises worldwide questions the scientific community about disaster management [1]. Besides technical skills, soft-skills, defined as *‘the cognitive, social, and personal resource skills that complement technical skills, and contribute to safe and efficient task performance’* [2]*,* have proved to be highly valuable when a disaster strikes [3]. Numerous studies have suggested that simulation may play a key role in soft-skills training [4]. Although disaster education is now a prerequisite in school curricula [5–7], very little is known about the content or teaching methods [8, 9]. We aimed to analyse primary and secondary school-based disaster education programs to identify which type of soft skills are taught and how.

**Materials and methods**

A literature research was conducted to find peer-reviewed articles, which were relevant to school-based disaster education up to December 2018. Data were then extracted and analysed according to Bloom’s skills classification [10] (Knowledge, Skills and Attitudes), the six skill components (e.g. people-related, reflexive, personal) and the teaching methods (teacher- or student-centred approach).

**Results**

Seven relevant articles were identified out of the 127 found. Soft-skills training was found in secondary school students in six studies. Of the seven studies, only one showed an integration of knowledge, skills and attitudes with all the soft-skill components. Regarding the latter, only three studies included reflexive and people-related skills. Concerning teaching methods, five studies used teacher-centred approach (lectures) to improve knowledge or skills, while simulation based-learning (student-centred approach) was found in one study, mainly to teach attitudes (e.g. leadership).

**Conclusions**

Soft-skills and simulation-based training are still largely underdeveloped in school-based disaster education. Existing initiatives remain too focused on classic teacher-centred approaches and basic or personal soft-skills knowledge. Further research is needed to develop more relevant soft-skills education tool for disaster medicine and to evaluate their efficacy.

**Acknowledgments**

We warmly acknowledge all the IKIC partners and Interreg-EMR organisation: Maastricht University, RWTH Aachen University, Order of Malta, School of the Landassociation North-Rhine e.V. (German Red Cross), Fire department of the City of Aachen, South Limburg Security Region, Research centre Jülich, Technical and Pedagogical Cooperation Centre and Hasselt University.

**References**

[1] Baubion C. OECD Risk Management: Strategic Crisis Management, *OECD Working Papers on Public Governance*, 2013;23

[2] Flin R, Maran N. Identifying and training non-technical skills for teams in acute medicine. *Qual Saf Health Care* 2004; 13: i80-4.

[3] Goralnick E, Van Trimpont F, Carli P. Preparing for the Next Terrorism Attack. *JAMA Surg* 2017; 152: 419.

[4] Morgan PJ, Kurrek MM, Bertram S, et al. Nontechnical Skills Assessment After Simulation-Based Continuing Medical Education. *Simul Healthc J Soc Simul Healthc* 2011; 6: 255–259.

[5] Izadkhah YO, Hosseini M. Towards resilient communities in developing countries through education of children for disaster preparedness. *Int J Emerg Manag* 2005; 2: 138.

[6] Johnson VA, Ronan KR, Johnston DM, et al. Evaluations of disaster education programs for children: A methodological review. *Int J Disaster Risk Reduct* 2014; 9: 107–123.

[7] Codreanu TA, Celenza A, Ngo H. Disaster Risk Education of Final Year High School Students Requires a Partnership with Families and Charity Organizations: An International Cross-sectional Survey. *Prehosp Disaster Med* 2016; 31: 242–254.

[8] Johnson VA, Fulbright New Zealand., Ian Axford (N.Z.) Fellowships in Public Policy. *Disaster preparedness education in schools : recommendations for New Zealand and the United States*. Fulbright New Zealand, 2011.

[9] Ronan KR, Crellin K, Johnston DM. Community readiness for a new tsunami warning system: quasi-experimental and benchmarking evaluation of a school education component. *Nat Hazards* 2012; 61: 1411–1425.

[10] Bloom BS. Taxonomy of educational objectives: the classification of educational goals*.* New York: Longmans, Green, 1956.

### A12 Crew Resource Management-study: results of a pre-post multicentric intervention study to improve teamwork in acute care settings

#### Leen Roes^1^, Sarah De Schepper^1^, Erik Franck^1,2^

##### ^1^Karel de Grote University College, Antwerp, Belgium; ^2^University of Antwerp, Antwerp, Belgium

###### **Correspondence:** Leen Roes (dieter.smis@kdg.be)

**Background**

Most adverse events in acute healthcare can be attributed to the poor non-technical skills of the teams. Team training, such as crew resource management, addresses these skills. The aim of this study was to 1) develop a generic Crew Resource Management (CRM)-training fit for all wards that regularly experience emergency situations, and 2) evaluate the training’s efficiency.

**Materials and methods**

Mixed method pre-post intervention study design was used to assess the participant’s satisfaction, learning and change in behavior, according to Kirkpatrick’s evaluation framework for training programs. Participants took part in CRM-based team training, which included 1) a theoretical part, and 2) a simulation part followed by debriefing. We used standardised questionnaires, observation and interviews. Fourteen wards (591 healthcare workers) were trained (5 obstetrics, 8 emergency and 1 intensive care unit).

**Results**

The participants valued the experience highly (mean 7.4/10 for satisfaction). The part of the survey testing for participant’s learning demonstrated a better understanding of non-technical skills. Observation showed significant improved teamwork skills (6.5 versus 5.5 on the Clinical Teamwork Scale, p=0.003). Safety attitudes measured with the Safety Attitudes Questionnaire were unaffected. The outcomes on these four levels did not differ between the different types of wards.

**Conclusions**

The strongest points of our study are the generic character, ‘in situ’ format of the training, the large sample size and the assessment of the training covering all four levels of Kirkpatrick. We found no change in safety attitudes. This may be related to the emphasis of the training on teamwork skills; other aspects such as job satisfaction may not have been affected. Furthermore, the timing of assessment immediately after training and the onetime CRM-training may have been insufficient for changing safety culture. The implementation of a CRM-based team training was well accepted and contributed to a significant improvement in teamwork skills in all acute care wards.

### A13 Simustress: how to adapt simulation sessions to allow skills development applied to stress management?

#### Frederic Senny^1^, Nathalie Dumont^2^, Laurence Peeters^2^, Mireille Appeltants^2^, Patrick Govers^3^, Thierry Robert^4^, Birgit Quinting^4^

##### ^1^Department of Industrial Engineering, HELMo-Gramme, Liège, Belgium; ^2^Department of Midwifery Nursing, HELMo-Sainte-Julienne, Liège, Belgium; ^3^Department of Social Sciences, HELMo-ESAS, Liège, Belgium; ^4^Department of Biotechnology, HELMo-Sainte-Julienne, Liège, Belgium

###### **Correspondence:** Frederic Senny (f.senny@helmo.be)

**Background**

Simulation practices are known to improve learning of technical, non-technical and communication skills. A manageable amount of stress is required for optimal learning (“stretch” zone) whereas a too high stress amount hinders learning process. The Simustress project aimed to assess students’ stress during simulation sessions in order to highlight its causes and to adapt simulation sessions accordingly.

**Materials and methods**

A mixed method approach was used. Data of 40 midwifery students in the third and fourth year of a nursing school are composed of four sets, one for each of the four simulation sessions (2016-10 to 2017-12). Data from continuous ECG recording, salivary cortisol, 3 surveys about the stress felt before, right-after, and at the end of the practice, transcriptions of several discussions with instructors and students separately were pre-processed, then imported in NVIVO software to be analysed quantitatively and qualitatively in order to point out the causes of stress. Students were previously informed and signed a written agreement.

**Results**

Complete data have been gathered for 29 students. ECG coding analysis reported a significant relative change in heart rate in more than 70% of the recordings during simulation and (de-)briefing. The results of the cortisol assay could not be interpreted with sufficient precision to contribute to the identification of stress sources. The stress surveys showed an average stress score of 5.6 (over 10) for the first session and of 4.4 in the fourth session. The surveys and the transcriptions allowed to identify six major themes of causes of stress: preparation of materials, artefacts of materials, novelty of a simulation session, rumours about a simulation session, instructors, and peers.

**Conclusions**

Stress is present in simulation and seems to be linked to modifiable causes. According to these results, the simulation practices were adapted in order to fit student’s stress to the “stretch” zone, through the enhancement of the communication and preparation before practices, and the emphasis on the respect of a good-behaviour chart.

